# A framework for identification and classification of liver diseases based on machine learning algorithms

**DOI:** 10.3389/fonc.2022.1048348

**Published:** 2022-10-14

**Authors:** Huanfei Ding, Muhammad Fawad, Xiaolin Xu, Bowen Hu

**Affiliations:** ^1^ The First Affiliated Hospital of Zhengzhou University, Zhengzhou, China; ^2^ School of Public Health and Second Affiliated Hospital, Zhejiang University School of Medicine, Hangzhou, China

**Keywords:** hepatocellular carcinoma, hepatitis B cirrhosis, classification, artificial intelligence, liver disease

## Abstract

Hepatocellular carcinoma (HCC) is one of the most commonly seen liver disease. Most of HCC patients are diagnosed as Hepatitis B related cirrhosis simultaneously, especially in Asian countries. HCC is the fifth most common cancer and the second most common cause of cancer-related death in the World. HCC incidence rates have been rising in the past 3 decades, and it is expected to be doubled by 2030, if there is no effective means for its early diagnosis and management. The improvement of patient’s care, research, and policy is significantly based on accurate medical diagnosis, especially for malignant tumor patients. However, sometimes it is really difficult to get access to advanced and expensive diagnostic tools such as computed tomography (CT), magnetic resonance imaging (MRI) and positron emission tomography (PET-CT)., especially for people who resides in poverty-stricken area. Therefore, experts are searching for a framework for predicting of early liver diseases based on basic and simple examinations such as biochemical and routine blood tests, which are easily accessible all around the World. Disease identification and classification has been significantly enhanced by using artificial intelligence (AI) and machine learning (ML) in conjunction with clinical data. The goal of this research is to extract the most significant risk factors or clinical parameters for liver diseases in 525 patients based on clinical experience using machine learning algorithms, such as regularized regression (RR), logistic regression (LR), random forest (RF), decision tree (DT), and extreme gradient boosting (XGBoost). The results showed that RF classier had the best performance (accuracy = 0.762, recall = 0.843, F1-score = 0.775, and AUC = 0.999) among the five ML algorithms. And the important orders of 14 significant risk factors are as follows: Total bilirubin, gamma-glutamyl transferase (GGT), direct bilirubin, hemoglobin, age, platelet, alkaline phosphatase (ALP), aspartate transaminase (AST), creatinine, alanine aminotransferase (ALT), cholesterol, albumin, urea nitrogen, and white blood cells. ML classifiers might aid medical organizations in the early detection and classification of liver disease, which would be beneficial in low-income regions, and the relevance of risk factors would be helpful in the prevention and treatment of liver disease patients.

## Introduction

Despite advances in both the diagnostic and management of patients with liver disease, the access for early diagnosis based on basic and cost effective clinical parameters like biochemical and blood routine test is frequently unavailable, which has a large impact on the clinical outcomes and quality of life for patients suffering from liver disease. In addition, it is really difficult to get access to advanced and expensive diagnostic tools such as computed tomography (CT), magnetic resonance imaging (MRI) and positron emission tomography (PET-CT)., especially for people who resides in poverty-stricken area and can’t afford these expensive tests.

Hepatocellular carcinoma (HCC) is the fifth most common cancer worldwide, and chronic HBV and HCV infection remains the major etiological contributor of HCC cases globally. WHO estimated that 257 million people (3.5% of the global population) are chronically infected with HBV infection as of the year 2015 (1, 2). The establishment of HBV chronicity depends on the age of exposure. The children younger than age 2 have 90% greater risk of HBV infection than adults (3, 4). Therefore, most chronic HBV infections are acquired vertically (i.e., mother to child transmission) at birth or feeding stage. Universal birth-dose HBV vaccination reduces the prevalence of chronic HBV in newborns (5); thus, most patients with chronic HBV were born in the pre-vaccination era.

Patients with chronic HBV are at risk of liver disease such as cirrhosis and HCC. The incidence of these liver diseases parallels the prevalence of chronic HBV (6), and therefore, the global distributions of chronic HBV and HCC mirror each other (7–10). It is estimated that chronic HBV is etiologically implicated in as many as 50% to 80% of all HCC cases, especially in HBV endemic areas (where chronic HBV prevalence is greater than 8%) (10, 11). The lifetime risk of chronic HBV carriers to develop cirrhosis and/or HCC is 15% to 40% (12, 13). The relative risk ratio of HCC in patients with chronic HBV ranged from 14 to 223 compared with that in noncarriers (14–16). The risk is substantially increased in those patients with liver cirrhosis (17). According to a systematic review in Asia, the incidence rates of HCC were 0.2, 0.6, and 3.7 per 100 person-years in inactive carriers, noncirrhotic chronic HBV, and cirrhotic chronic HBV, respectively (18).

The diagnosis of liver disease or condition depends on the information that includes risk factors that make accurate diagnosis difficult. These risk factors include resource and organizational limits, conflicts, ambiguity, and uncertainty. Many symptoms are vague and vary from person to person. Several diagnostic tests are costly, seldom performed, and often do not provide a black-and-white result. In addition, cognitive bias and improper use of heuristics are common occurrences during the diagnostic phase among physicians. This study intends to present a framework to aid medical professionals and other interested researchers in properly diagnosing liver disease, utilizing comprehensive assessment criteria. The outcomes of this research will aid physicians in making more precise judgments about liver disease identification.

## Materials and methods

### Patients

The study population consisted of 525 retrospectively reviewed consecutive patients who were suspected to have liver disease at The Affiliated Hospital of Zhengzhou University. We demonstrated the performance of the selected classifier models on the 525 patient’s liver disease data. The 525 patients’ liver disease data are divided into training and validation subsets. The training sample was used to train a model, and validation was used for model testing. In addition, we compare the classifier models using statistical measures to obtain the best classifier model. Based on the best classifier model, we identified significant factors contributing to liver disease. All results were performed in the R programming language.

### Regularized regression (RR) classifier

RR is a classification approach that uses a penalized regression with coefficient estimates biased towards zero to regress the category of interest on text features. In other words, to avoid the problem of overfitting, this approach prevents us from learning a more complex or flexible model. Ridge, lasso, and elastic net are frequently used penalty parameters for RR. The elastic net is a generalization of the ridge and lasso penalties that combines the two penalties used in the present study (19). The elastic net technique learns from the shortcomings of the lasso and ridge regression methods to improve the RR classifier.

### Logistic regression (LR) classifier

The Logistic classifier algorithm is based on LR (20) and used to determine the relationship between a categorical target variable and several input variables. To make predication, as with inputs, it requires actual values based on the probability of an input belonging to a particular class. The probability is determined using a sigmoid function that incorporates the exponential function. LR is frequently used in machine learning due to its high efficiency and low computing resource requirements (21).

### Decision tree (DT) classifier

The DT aims to develop a model that predicts the target variable by learning simple decision rules inferred from the input features. DT is the form of a tree that consists of a series of decisions and choices. They determine the class of a variable based on its features. Generally, these classes reside on the last branches of a DT. It may be binary or multi-class classifiers. Multiple rules with binary outcomes are used to build a set of tests that determine the class of a variable based on its features. DTs are an example of a divide and conquer method since they split the variables repeatedly until a final decision is reached (22). Due to their understandability and simplicity, DT is one of the most popular machine learning algorithms (23).

### Random forest (RF) classifier

RFs, also known as random decision forests, consist of many random DTs. RFs developed a model using a random sampling of the actual data and a random subset of features (24). This randomness contributes to the model being more resilient than a DT and less prone to overfitting on the training data (25). Each tree provides a classification, which we call a “vote” for that class. The classification with the highest votes is chosen by the forest (over all the trees in the forest) (26).

### Extreme gradient boosting (XGBoost) classifier

XGBoost offers a parallel tree-boosting approach that efficiently and precisely addresses various data science issues. The Extreme Gradient boost is a powerful and scalable classification algorithm developed by Chen and Guestrin (27). The fundamental concept of Extreme Gradient boost is the gradient boosting decision tree, which produces the final predictions from the combined prediction of many DTs. In Extreme Gradient boost, each new tree is constructed sequentially, attempting to correct the errors of the previous trees until the number of DTs specifies the threshold.

### Performance measures for classification algorithm

After completing the training phase, all the models were tested on the test dataset. The performance of classification algorithms is evaluated through statistical indicators. This study uses four performance indicators: accuracy, precision, recall, F1-score, and area under curve (AUC). The performance measurements utilized in this article are detailed in the following section. Accuracy indicates how closely the measured value corresponds to a known value, while precision provides the accuracy of the model in terms of those predicted to be positive. A recall defines the number of real positives the model collected after being labeled as positive (true positive). The F1 provides a ratio between precision and recall. The receiver operating characteristics (ROC) is a probability curve, whereas AUC reflects the degree of separability. The ROC curve represents the relationship between sensitivity (true positive rate) and specificity (false positive rate) (28, 29).

## Results

**Figure 1:**
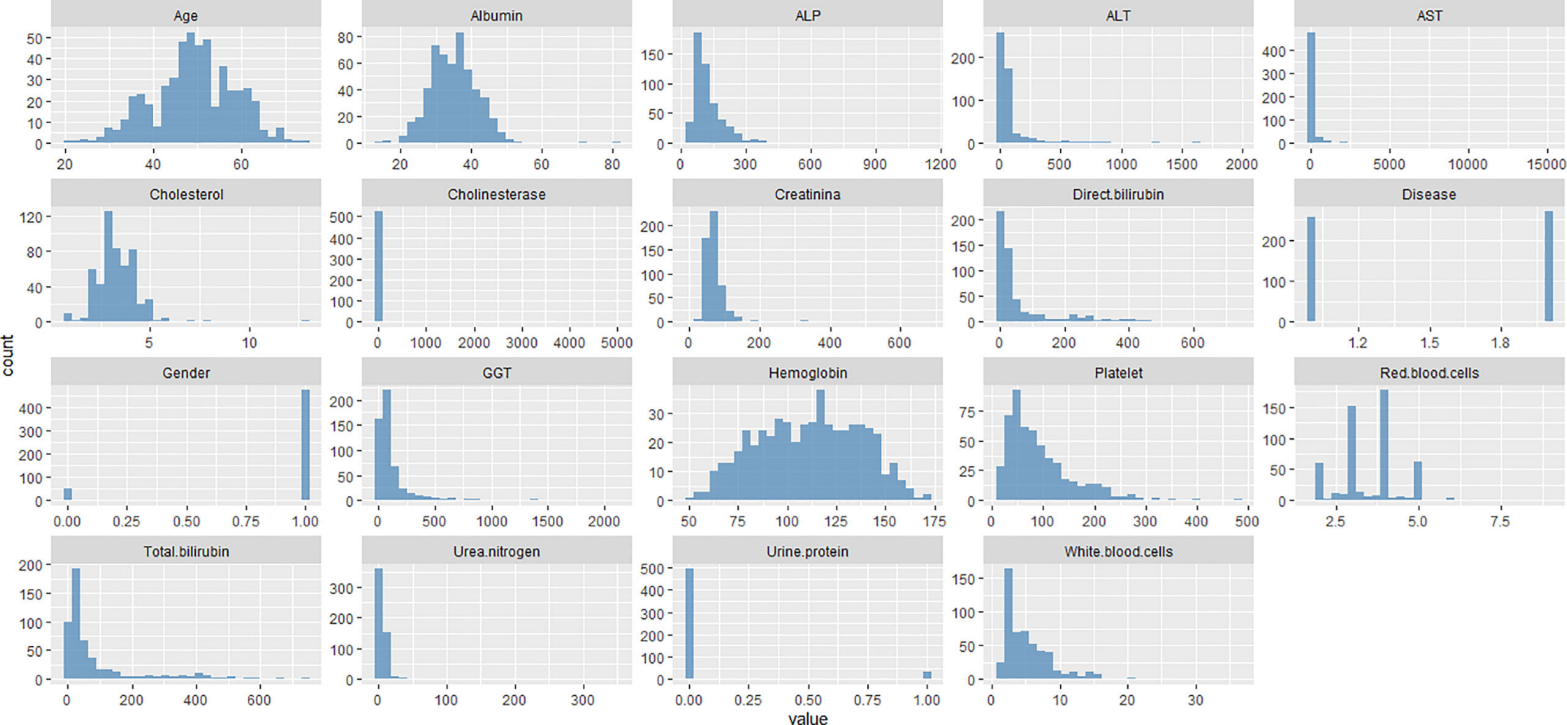
Depicts the visual inspection of patient characteristics used in the study.

The collected parameters of liver disease patients included age, gender, albumin, ALP, ALT, AST, total bilirubin, cholinesterase, cholesterol, creatinine, GGT, urine protein, white blood cells, red blood cells, hemoglobin, platelet, direct bilirubin, and urea nitrogen used in the study are displayed in **Figure 1**. The study consists of 472 male and 53 female patients with an average age of 49.31 years. Among the 525 patients with liver disease, 256 had Hepatitis B cirrhosis, and 269 had Hepatocellular carcinoma.

The findings of the classifier models for testing data based on performance metrics are provided in **Table 1**. The results highlighted in bold emphasize that a particular classifier model performed significantly better than the other classifier models. The RF classifier obtains the highest accuracy (0.762) among all classifier models, while the RR classifier has the second highest accuracy (0.743). However, the RR classifier had the highest precision (0.731), and the LR classifier had the second-highest precision (0.725). According to **Table 1**, the classifiers with the best performance in terms of recall are RF, followed by DT. It is also observed from **Table 1** that the RF classifier yields the best results in terms of F1-score and AUC, followed by RR and XGBoost classifiers, respectively. Overall, the findings based on performance metrics reveal that the RF has the best classier model for liver disease.

**Table 1:** Performance measure of classifier machine learning models.

Model	Accuracy	Precision	Recall	F1-score	AUC
Regularised regression	0.743	0.731	0.745	0.738	0.815
Logistic regression	0.733	0.725	0.725	0.725	0.819
Random forest	0.762	0.717	0.843	0.775	0.999
Extreme Gradient boosting	0.724	0.704	0.745	0.724	0.989
Decision tree	0.657	0.615	0.784	0.690	0.878

**Figure 2:**
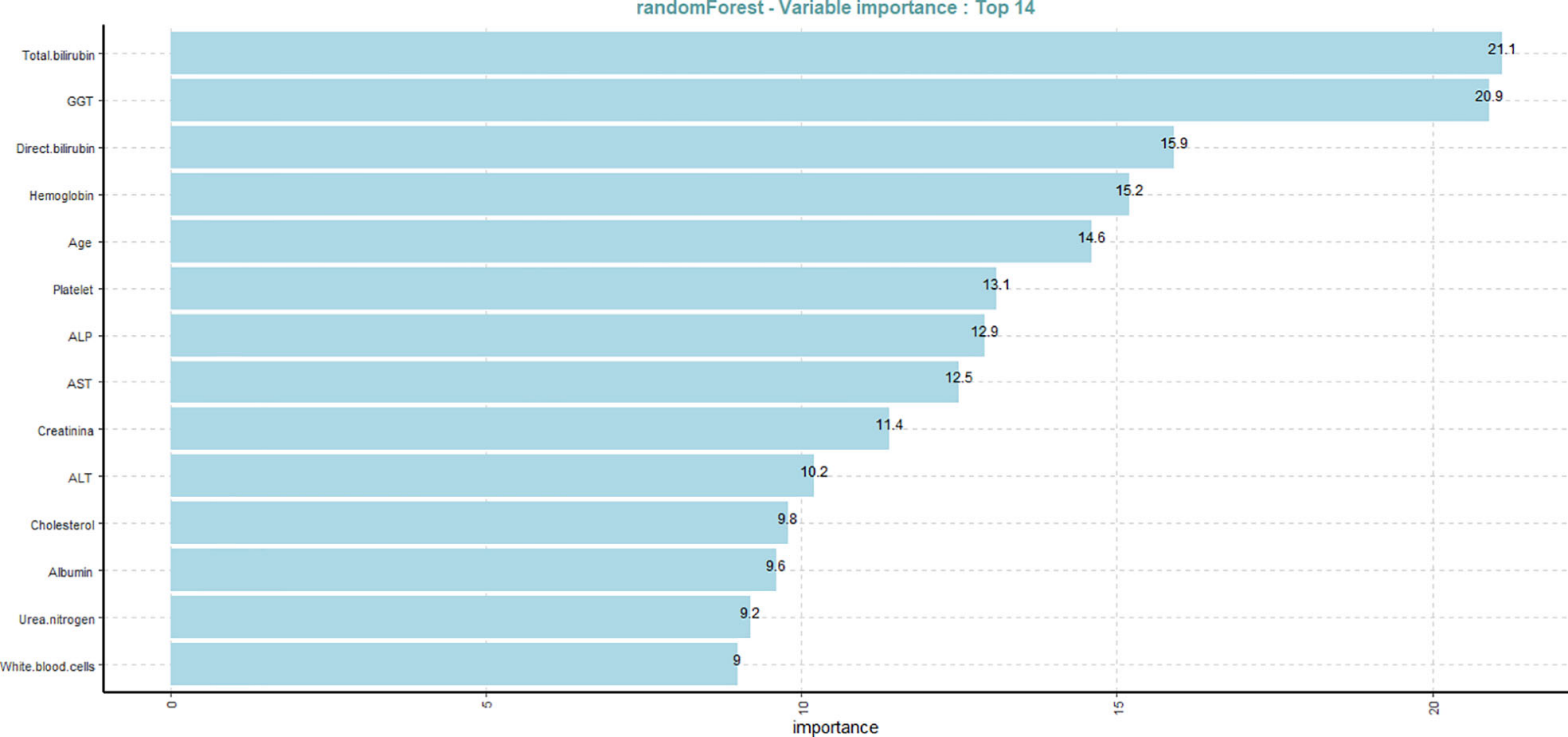
Variable importance plot of the 14 risk factors of liver disease based on the random forest classier.

After finding the optimum classier model, next we determine the variables importance based on the RF model, as presented in **Figure 2**. Variable importance describes the degree to which a model utilizes a certain variable to generate accurate predictions. Variables of high importance are drivers of the output, and their values have a substantial effect on the output values, while variables with little relevance may be excluded from a model to make it easier and quicker to fit and predict. Based on the mean decrease Gini and clinical experience using the RF algorithm, the final 14 important variables are Total bilirubin, GGT, direct bilirubin, hemoglobin, age, platelet, ALP, AST, creatinine, ALT, cholesterol, albumin, urea nitrogen, and white blood cells.

## Discussion

HCC is the most common liver disease. HCC etiologies vary based on geography, lifestyle, and advanced medical care facilities availability. Although, NAFLD/NASH and excessive alcohol intake are the important risk factors leading to the development of HCC. However, at present, most of the HCC cases are caused by chronic infection from HBV or HCV (30). With the rising rates of obesity and diabetes mellitus as well as the declining levels of alcohol intake and viral hepatitis infection in many areas, It is expected that NAFLD/NASH will become the most important risk factor for HCC (31). Consequently, it is crucial and should be emphasized on surveillance and early diagnosis of HCC in at-risk populations. Universal unified prevention measures, education on high-risk behaviors, and screening programs for blood donors are crucial to prevent and reduce HBV and HCV induced HCC. However, vaccination is the key to prevent HBV-related HCC. Current antiviral therapies for HBV and HCV infection can only decrease HCC but cannot entirely eradicate it (32). Treatment of HCC has improved substantially over the last decades, with several curative options. Novel therapies, such as radioembolization with 90Y-labeled glass beads, and medications, such as sorafenib and regorafenib, have shown improvements in survival rates. However, there are serval areas where still improvement is needed (33). Additional studies are needed to improve prevention strategies and advance management of patients with HCC, especially in the field of tumor regression therapies.

In 2015, nearly 1 million persons died because of complications of chronic HBV. In contrast to the reductions in mortality from other important infections, such as HIV, tuberculosis, and malaria, the mortality from chronic HBV-related complications increased over the past decade (1). Specifically, HBV cirrhosis-related deaths were 241,700 in 1990 compared with 312,400 in 2010. The numbers of deaths from chronic HBV-related HCC were 210,200 in 1990 compared with 341,400 in 2010. Deaths from HBV-associated HCC occur at a younger age in sub-Saharan Africa (median age 38.9) than in the Western Pacific region (median age 54.5) (34).

Even though several risk factors were etiologically illustrated for HCC, there are still dead zone in this field in terms of the un-satisfactory prognosis for HCC. For the reason that sometimes patients cannot get access to advanced and costly diagnostic tools such as CT, MRI and PET-CT etc., especially for people who has the problem of economics. Therefore, the results of our study imply a framework for early diagnosis of liver diseases based on basic tests like biochemical and blood routine test, which is easily accessible for almost all people all over the word. This study used more powerful machine learning classifier models with 18 liver disease parameters filtrated by clinical experience. The classifier models were compared based on the accuracy, precision, recall, F1-score, and AUC measures. We found that the RF classifier outperformed RR, LR, extreme gradient boosting, and DT in terms of accuracy, recall, F1-score, and AUC measures. In contrast, the RR classifier achieved the highest precision among all other classifier models. Overall, the findings of this research demonstrate that the RF model performed the best for the classification of liver disease. Furthermore, the variable importance plot revealed the most significant clinical factors that contributed the most to liver disease based on the RF model. Further, including other risk factors might also aid classification algorithms in accurately identifying liver disease in a patient. Studies conducted based on various age groups and topologies might assist in highlighting the contributions of several risk factors in the identification of liver disease. Future work should concentrate on these areas in order to increase model accuracy.

## Conclusion

In this study, we applied ML classification algorithms to accurately detect liver disease in 525 with 18 risk factors filtered by clinical experience. The performance of ML classification algorithms is determined based on accuracy, precision, recall, F1-score, and AUC measures. Our findings show that the RF classifier model is the best-performing algorithm for liver disease classification among all RR, LR, DT, and XGBoost classifiers. Further, we identified the 14 important risk factors based on the RF classifier that has contributed the most to liver disease in 525 patients. The outcomes of this study will aid medical professionals and researchers in making better conclusions about identifying liver disease, which could also guide or improve the treatment of patients who do not have a clinical diagnosis.

## Data availability statement

The raw data supporting the conclusions of this article will be made available by the authors, without undue reservation.

## Ethics statement

The studies involving human participants were reviewed and approved by The First Affiliated Hospital of Zhengzhou University. The patients/participants provided their written informed consent to participate in this study.

## Author contributions

Study Concept and Design: MF, HD, and XX; Manuscript Writing: MF, HD, and BH; Data Collection: BH; Analysis of Data: MF and XX; Critical revision of the Manuscript: BH, HD, and XX. All authors contributed to the article and approved the submitted version.

## Funding

This study was financially supported by grants from Hepatobiliary foundation of Henan Charity General Federation (No: GDXZ2022003) and Hepatobiliary foundation of Henan Charity General Fedration (No: GDXZ2019006).

## Conflict of interest

The authors declare that the research was conducted in the absence of any commercial or financial relationships that could be construed as a potential conflict of interest.

## Publisher’s note

All claims expressed in this article are solely those of the authors and do not necessarily represent those of their affiliated organizations, or those of the publisher, the editors and the reviewers. Any product that may be evaluated in this article, or claim that may be made by its manufacturer, is not guaranteed or endorsed by the publisher.
